# Intrinsic tumor necrosis factor-α pathway is activated in a subset of patients with focal segmental glomerulosclerosis

**DOI:** 10.1371/journal.pone.0216426

**Published:** 2019-05-16

**Authors:** Chen-Fang Chung, Thomas Kitzler, Nadezda Kachurina, Katarina Pessina, Sima Babayeva, Martin Bitzan, Frederic Kaskel, Ines Colmegna, Nada Alachkar, Paul Goodyer, Andrey V. Cybulsky, Elena Torban

**Affiliations:** 1 Department of Medicine, McGill University and McGill University Health Center, Montreal, QC, Canada; 2 Department of Paediatrics, McGill University and McGill University Health Center, The Montreal Children’s Hospital, Montreal, QC, Canada; 3 Department of Pediatric Nephrology, Albert Einstein College of Medicine, New York, NY, United States of America; 4 Department of Medicine, The Johns Hopkins University School of Medicine, Baltimore, MD, United States of America; University of Houston, UNITED STATES

## Abstract

Focal segmental glomerulosclerosis (FSGS) is frequently found in biopsies of patients with steroid resistant nephrotic syndrome (SRNS). The pathogenesis of SRNS/FSGS is often unknown and the disease will recur in up to 50% of patients post-transplant, indicating the presence of circulating podocyte-toxic factor(s). Several studies have reported clinical improvement after anti-TNFα therapy. However, prediction of the clinical outcome in SRNS/FSGS is difficult, and novel predictive biomarkers are needed. An image-based assay, which measures disassembly of focal adhesion complexes in cultured podocytes, was used to ascertain the presence of podocyte toxic activity in SRNS/FSGS sera. Expression of TNFα pathway genes was analysed in the Nephroseq FSGS cohort and in cultured podocytes treated with SRNS/FSGS sera. Podocyte toxic activity was detected in 48/96 SRNS/FSGS patients. It did not correlate with serum TNFα levels, age, sex, ethnicity or glomerular filtration rate. In ~25% of the toxic samples, the toxicity was strongly inhibited by blockade of TNFα signaling. Transcriptional profiling of human FSGS biopsies and podocytes treated with FSGS sera revealed significant increases in expression of TNFα pathway genes. We identified patients with serum podocyte toxic activity who may be at risk for FSGS recurrence, and those patients in whom serum podocyte toxicity may be reversed by TNFα blockade. Activation of TNFα pathway genes occurs in podocytes of FSGS patients suggesting a causative effect of this pathway in response to circulating factor(s). *In vitro* analyses of patient sera may stratify patients according to prognostic outcomes and potential responses to specific clinical interventions.

## Introduction

In the majority of pediatric and in a significant proportion of adult patients who present with sudden onset of the nephrotic syndrome, proteinuria resolves completely with administration of steroid therapy. However, approximately 10–15% of children and a greater percent of adults are resistant to steroids (steroid-resistant nephrotic syndrome, SRNS) [1]. In these patients, renal biopsies commonly show focal and segmental glomerular sclerosing lesions (FSGS) [2]. FSGS lesions reflect an irreversible change in the glomerulus due to podocyte loss that results in adhesions between the denuded capillary and Bowman’s capsule. Many patients with FSGS progress to end stage renal disease, necessitating dialysis and renal transplantation. In up to 50–70% of children under 1 year of age at the onset, and ~20% of children older than 1 year and adults with SRNS/FSGS, the disease can be attributed to mutations in genes that encode key proteins in the podocyte [3–6]. In the absence of such mutations, SRNS/FSGS can occasionally be attributed to viruses (e.g. HIV) [7] or nephrotoxic drugs [8, 9]. However, in the majority of SRNS/FSGS patients without identifiable genetic mutations, the disease etiology is “idiopathic”. Indeed, FSGS most likely is not a single disease entity, and diverse molecular mechanisms may contribute to the pathogenesis. Importantly, patients with idiopathic SRNS/FSGS are at ~50% risk for recurrent disease in the allograft after renal transplantation [4]. This constitutes strong evidence that renal disease is driven by an extrarenal podocyte-toxic factor that is likely produced by malfunctioning immune cells [10, 11].

Over the last three decades, several candidate molecules have been proposed as SRNS/FSGS causative factors; however, to date, none of them has been confirmed as the primary cause of the disease [12, 13]. Difficulties in identification of the SRNS/FSGS podocyte toxic factor likely reflect the heterogeneity of the SRNS/FSGS patient cohorts and the lack of reliable test systems to detect the podocyte toxic activity in serum. Prior to transplantation, it is often unclear whether an individual patient with idiopathic SRNS/FSGS harbors a mutation in an undiscovered podocyte gene or whether there is high risk of recurrence post-transplant, making prognostication of the outcome unpredictable.

Several reports suggest a role for tumor necrosis factor (TNF)-α pathway in the pathogenesis of SRNS/FSGS. Increased levels of plasma or urine TNFα [14, 15] and elevated urinary excretion of soluble TNFα Receptor I and plasma concentrations of soluble TNFα Receptors I and II were reported in FSGS patients [16, 17]. A rapid improvement of severe nephrotic syndrome in a patient with autosomal-dominant TNFα Receptor–associated periodic syndrome occurred upon administration of recombinant human TNFα Receptor I-Fc fusion protein (etanercept) [18]. The periodic syndrome is caused by mutations in the TNFα Receptor I and is associated with lifelong febrile attacks of abdominal and musculoskeletal pain. A biopsy was not performed and, thus, the type of glomerular injury underlying the nephrotic syndrome is unknown. In another patient with recurrent FSGS, proteinuria was alleviated by infliximab, a monoclonal chimeric antibody against TNFα, as well as etanercept [19]. Likewise, in the first phase of the Novel Therapies for Resistant FSGS, FONT, study, four out of 10 SRNS/FSGS patients achieved at least 50% reduction in proteinuria in response to anti-TNFα antibody, adalimumab [20]. However, the subsequent FONT II study reported no improvement among seven SRNS/FSGS patients treated with adalimumab [21], suggesting significant heterogeneity among patients in response to anti-TNFα therapy.

We previously reported a patient with recurrent FSGS whose plasmapheresis effluent caused a dramatic disassembly of polymerized actin in cultured human podocytes [22]. These toxic effects *in vitro* could be rescued by incubation of cultured podocytes with etanercept, adalimumab or with a mix of blocking antibodies against TNFα Receptor I and II [22]. The effect of this patient’s serum on cultured podocyte cytoskeleton was similar to that of recombinant TNFα *in vitro* [22, 23]. Treatment of the patient with infliximab reduced proteinuria and allowed discontinuation of plasmapheresis therapy [22]. Collectively, these observations raise the possibility that the TNFα signaling pathway may be involved in the pathogenesis of nephrotic syndrome in some (but not all) idiopathic SRNS/FSGS patients. However, there is currently no clinical algorithm to identity individual SRNS/FSGS patients who may benefit from TNFα blockade.

Recently, we developed a computerized image-based *in vitro* assay to test for the presence of podocyte-toxic factors in patient serum by analyzing the effect of serum on the integrity of podocyte focal adhesion complexes (FACs) [23]. Sera from patients with recurrent FSGS consistently dispersed podocyte FACs *in vitro*, reducing FAC number to below 60% of untreated cells within 20 h. In contrast, sera from SRNS/FSGS patients with podocyte gene mutations or those who did not recur post-transplant had no significant effect [23, 24].

In the present study, we used our podocyte FAC assay to screen 96 SRNS/FSGS serum samples to identify the subset of patients who display podocyte toxicity, estimate the percentage in whom this can be reversed *in vitro* by TNFα blockade, and ascertain whether podocyte toxicity correlates with the serum TNFα level. We demonstrate that 50% of the sera exert significant podocyte toxicity *in vitro* that was not associated with sex, age, estimated glomerular filtration rate (eGFR) or proteinuria. The toxic effect of SRNS/FSGS sera is independent of serum TNFα level or response to TNFα blockade. Using the Nephroseq database, we demonstrate that TNFα pathway gene expression is activated in glomeruli from SRNS/FSGS patients. Remarkably, the SRNS/FSGS sera exhibiting podocyte toxicity in the FAC assay triggered similar changes in podocyte TNFα gene expression *in vitro*. Therefore, serum from idiopathic SRNS/FSGS patients activates the intrinsic podocyte TNFα pathway and this is unrelated to circulating TNFα levels.

## Materials and methods

Ethics approvals have been obtained from the Institutional Review Boards of the McGill University and McGill University Health Center Research Institute. Written consent was obtained from each participant including healthy volunteers.

### Patient samples

This is a multi-center study using samples from SRNS/FSGS patients at the McGill University Health Center, MUHC (Montreal, Canada), Johns Hopkins Hospital, JHH (Baltimore, USA) and the Focal Segmental Glomerulosclerosis clinical trial (FSGS-CT, National Institute of Diabetes and Digestive and Kidney diseases, USA). The study at the MUHC was done with Institutional Review Board approval A07-M75-13A. Serum samples from the FSGS patients, patients with active RA and from healthy volunteers were collected with written informed consent from each participant. Blood collection and handling was according to standard procedures.

### Human podocyte cultures

Immortalized thermo-sensitive human podocytes (clone AB8/13, kindly provided by Dr. Moin Saleem, University of Bristol, UK [25]) were grown as previously described [23, 26]. For the *in vitro* toxicity assay, the cells were plated on coverslips coated with 0.01% human collagen I in PBS (Sigma-Aldrich, Oakville, CA), in 12-well plates (Sarstedt, Montreal, QC, CA) and grown at 33°C for 3 days until ~65% confluence. Then, the cells were allowed to differentiate for 14 days at 37°C; the medium was changed twice a week. Differentiated podocytes were treated for 20 h with 10% human serum (from either FSGS patients, RA patients or healthy controls) in medium without FBS. Within each experiment, treated podocytes were compared to control (untreated podocytes grown in 10% FBS) in the same 12-well plate. After treatment, cells were washed in PBS and fixed in 4% paraformaldehyde for 15 min.

### Immunostaining and analysis of podocyte toxicity and TNFα blockade sensitivity

Immunostaining and analysis of serum toxicity were carried out as described previously [23]. FACs were detected with anti-vinculin antibody (1:400 V9131, Sigma-Aldrich, St-Louis, MO, USA) followed by rabbit anti-mouse AlexaFluor-488 antibody (Molecular Probes, Eugene, OR, USA). Cell nuclei and cell shape were visualized by staining with 4′,6-diamidino-2-phenylindole (Invitrogen, Carlsbad, CA, USA) and Phalloidin-AlexaFluor-564 (Molecular Probes), respectively. ImageJ software [27] was used for cell analysis. To block TNFα signaling, podocytes were pre-incubated with a mix of monoclonal antibodies to TNFα receptor I (TNFRI; 0.25 μg/mL, MAB225, R&D Systems, Minneapolis, MI, USA) and TNF receptor II (TNFRII; 0.1 μg/mL, MAB226, R&D Systems) for 30 min prior to addition of patient sera, as described previously [22]. Images of control (untreated) cells and cells exposed to sera were acquired on the same 12-well plate under identical conditions. The experiments with the FSGS-CT samples were repeated once in duplicate for each condition; for all other samples, the experiments were repeated at least twice in duplicate for each experimental condition.

### TNFα ELISA

Measurement of the circulating TNFα concentration in all serum samples was done using the Human TNF-alpha Quantikine ELISA Kit (R&D Systems, Minneapolis, MI, USA) according to the manufacturer’s recommendations. All experiments were done in triplicate with the serum samples diluted in half. Measurements for the FSGS-CT samples were done once in triplicate; for all other samples, the ELISA detection was repeated twice in triplicate. The detection threshold of the ELISA kit is 4 pg/ml.

### Nephroseq dataset analysis

The publicly accessible Nephroseq dataset was used for the expression analysis of glomerular TNFα signaling pathway genes [28]. The TNFα pathway gene query was created by combining the TNFα pathway-related genes listed in the KEGG pathway [29] and in the Qiagen human TNFα signaling pathway PCR Array kit (PAHS-063Z, Qiagen Canada, Montreal, QC, CA) (total of 157 genes). Principal component analysis (PCA) of TNFα signaling pathway gene expression (algorithm that identifies the maximal variations in the data and reduces the dimensionality to a few components) was employed [30].

### Quantitative polymerase chain reaction

Differentiated podocytes were treated with either FSGS or healthy sera, as above. RNA was extracted using Trizol (Sigma-Aldrich, Oakville, ON, CA). Reverse transcription was performed using 1 μg of RNA with Superscript III polymerase (ThermoFisher Scientific, Burlington, ON, CA). qPCR amplification was done using LightCycler 480 SYBR Green I Master mix (Roche Canada, Montreal, QC, CA) on a CFX384 quantitative PCR System (BioRad, Hercules, CA, USA). Amplification parameters and primers are listed in S1 Table. Five serum samples from healthy individuals and five FSGS sera were used. For each serum, two differentiated podocytes cultures were treated and RNA extracted (biological replicates). All experiments were done twice in triplicate for each biological replicate.

### Statistical analysis

Excel and GraphPad Prizm software were used for statistical analyses. One-way ANOVA was used in experiments in which more than 2 groups were compared, followed by a post-hoc t-test analysis (with Bonferroni correction).

## Results

### Screening FSGS patients for toxicity to cultured human podocytes

We screened sera from 96 SRNS/FSGS patients (Tables 1–3) with our podocyte FAC number assay [23]. Only 8 of 17 tested patients from the McGill University Health Centre (MUHC) cohort had pathogenic mutations in one of the known SRNS/FSGS genes [24]. In others, podocyte gene status is unknown. The MUHC and Johns Hopkins Hospital (JHH) patients included 22/27 patients who had received a renal allograft prior to blood collection; 7 of these patients had recurrent SRNS/FSGS in the allograft. Serum samples from healthy individuals and patients with active rheumatoid arthritis (RA) were used as controls (Table 4).

**Table 1:** Summary of the FSGS patient cohort.

**Parameters**	**FSGS Patients**
Total number	96
FSGS-CT cohort	69
MUHC cohort	17
JH cohort	10
**Age at diagnosis**	18 ± 12
**Age at blood collection (years, mean ± SD)**	22 ± 13
**Females/Males**	40/56
**Race/ethnicity**	
White	44/96 (46%)
African American	30/96 (31%)
Asian	2/96 (2%)
Hispanic	13/96 (14%)
American Indian	3/96 (3%)
other	4/96 (4%)
**UPCR, g/g**	5 ± 5*
**eGFR (ml/min/1.73 m^2^) at** **blood collection**	86 ± 51**

* The UPCR was available for 89 patients

**18 years and older: CKD-EPI equation (2009); for patients <18: Schwartz formula

**Table 2:** Detailed clinical characteristic of the patient cohorts used in the study.

Parameters	Clinical Characteristics		
	FSGS-CT* cohort	MUHC** cohort	Johns Hopkins Hospital cohort
**Number of patients**	69	17	10
**Females/males**	28/41	6/11	6/4
**Age at FSGS onset (years, mean ± SD)**	18 ± 10	8 ± 12	30 ± 15
**Age at blood collection (years, mean ± SD)**	20 ± 10 years	18 ± 12	44 ± 12
**Primary FSGS confirmed on biopsy**	^α^Light microscopy	Light/EM microscopy	Light/EM microscopy
**Urine protein/creatinine ratio, g/g**	5 ± 4	10 ± 10	2 ± 2
**Median eGFR at blood collection**	101 ± 59 ml/min/1.73 m^2^101	61 ± 56 ml/min/1.73 m^2^	31 ± 17 ml/min/1.73 m^2^
**Genetic status (%)**	Not available	8/17 (47%) patients bear pathogenic mutations in podocyte genes^β^	Not tested
**Kidney transplantation (%)**	none	12/17(71%)	10/10 (100%)
**Recurrence post-transplant (%)**	none	5/12 (30%)	2/10 (20%)
**Immunosuppression**	MMF^γ^, CSA^δ^	MMF, CSA, Tacrolimus (only for transplanted patients)	Prednisone, Tacrolimus

*FSGS-CT cohort–FSGS clinical trial cohort at the NIDDK

** MUHC- McGill University Health Center cohort

^α^Light microscopy: a minimum of 1 glomerulus demonstrating segmental sclerosis on light microscopy was required to confirm the diagnosis.

^β^Association of genetic mutations and toxicity on the Focal Adhesion Complex assay are described in Kitzler et al, PedNeph, 2018

^γ^MMF—mycophenolate mofetil

^δ^CSA -cyclosporin

**Table 3:** Inclusion and Exclusion Criteria for the FSGS-CT cohort.

FSGS-CT cohort	
Inclusion Criteria	Exclusion Criteria
**Age 2–40 years at onset of FSGS**	Secondary FSGS
**Primary FSGS confirmed on biopsy (all subtypes)**	Blood pressure > 140/95 or > 95^th^ percentile for age/height
**Steroid resistance for 4 weeks (no complete remission of proteinuria subsequent to 4-week course demonstrating steroid resistance)**	Use of anti-hypertensive medication
**> 1.0 g urinary protein/g creatinine on first am void**	Malignancy
**Estimated GFR ≥ 40 ml/min/1.73 m^2^**	GI and liver disease
**Native kidneys**	Active/serious infection (including, but not limited to Hepatitis B or C, HIV)
	Diabetic mellitus Type I and II
	Organ transplantation
	Obesity defined as i. BMI > 97th percentile for age if age 2-20 years ii. BMI > 40 kg/m^2^ for age ≥21 years
**Provided consent**	Inability to consent

Note: Genetic status of the FSGS-CT cohort is unavailable/unspecified

**Table 4:** Healthy controls and patients with rheumatoid arthritis (RA).

MUHC healthy volunteers	
Parameters	Characteristics
**Number of participants**	9
**Age (years, mean ± SD)**	35 ± 16
**Females/males**	7/2
**Consent**	yes
**RA patients**	
**Number of patients**	15
**Age (years, mean ± SD)**	47 ± 14
**Female/Male**	15/0
**Disease duration (years, mean ± SD)**	3.4 ± 6.9
**^α^RF positive**	12/15 (80%)
**^β^CCP positive**	11/15 (69%)
**Erosive disease**	5/15 (33%)
**^γ^DMARD**	7/15 (47%)
**Consent**	yes

Notes: all patients had active disease defined as a disease activity score (DAS28) higher than 5.1. Two of the patients had Sjogren syndrome

^α^RF–rheumatoid factor

^β^CCP–cyclic citrullinated peptide antibody

^γ^DMARD–disease-modifying antirheumatic drugs

**Fig 1:**
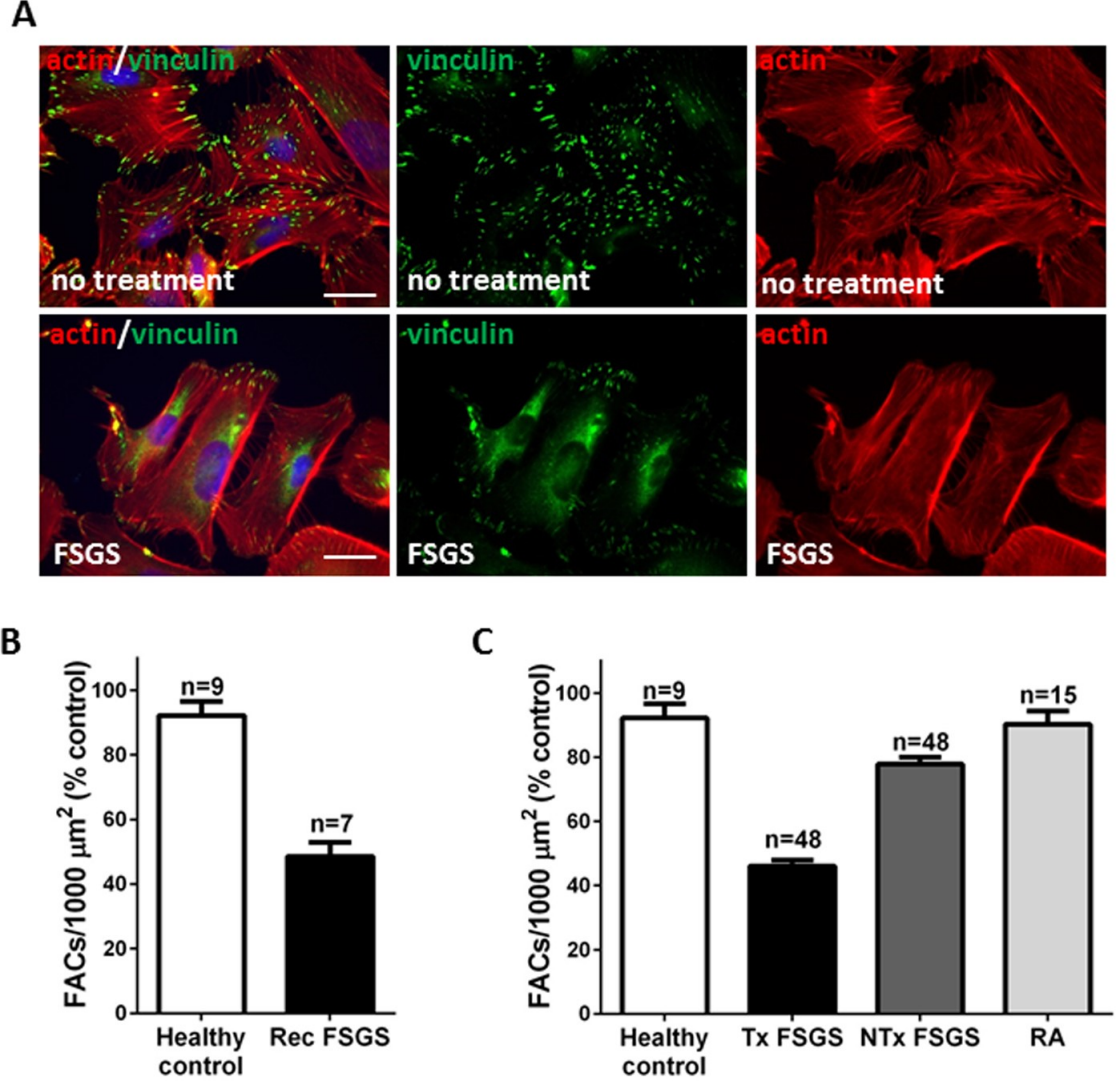
Analysis of the effects of human sera on focal adhesion complex integrity in cultured podocytes. (A) **Upper panels**—images of untreated podocytes grown in 10% FBS. Focal adhesion complexes were immunostained with anti-vinculin antibody (green), actin was visualized with phalloidin (red) and nuclei were stained with DAPI. **Lower panels**–images of podocytes treated with 10% serum from a patient with podocyte toxic activity (Tx); scale bar 5 μm. (B) FAC numbers after podocyte treatment with sera from healthy controls and patients with recurrent (Rec) FSGS (means and standard errors). (C) FAC numbers after podocyte treatment with sera from the SRNS/FSGS cohort, healthy volunteers and patients with rheumatoid arthritis (RA). The FAC numbers in the untreated podocytes grown in 10% FBS were calculated as 100%; the percentage of FACs in the cells exposed to human sera were compared to the untreated podocytes. NTx, non-toxic (means and standard errors).

Sera from all recurrent (allograft) SRNS/FSGS cases decreased FAC number to <60% of untreated cells (mean 47.1%, this paper) and [23]; FAC number in untreated cells was set at 100% (Fig 1A and 1B and [23]). Since recurrence of SRNS/FSGS in the renal allograft is in keeping with a circulating podocyte-toxic factor, we assigned a FAC number ≤60% as the *threshold* for a positive result (presence of podocyte toxicity) in our assay. On this basis, 50% (N = 48/96) of all FSGS sera reduced FAC number below the threshold (mean 45.9%) (Fig 1C). Mean FAC number for the remaining 50% of sera was 77.8% of untreated (Fig 1C). There were no significant differences in the number of FACs between cells treated with sera from healthy individuals (mean 90.3%) or from patients with RA (mean 92.2%), compared to untreated cells (Fig 1C). Taken together, these results confirm that the podocyte-toxic activity is unambiguously associated with the sera from the FSGS patients, but not RA or healthy sera.

**Fig 2:**
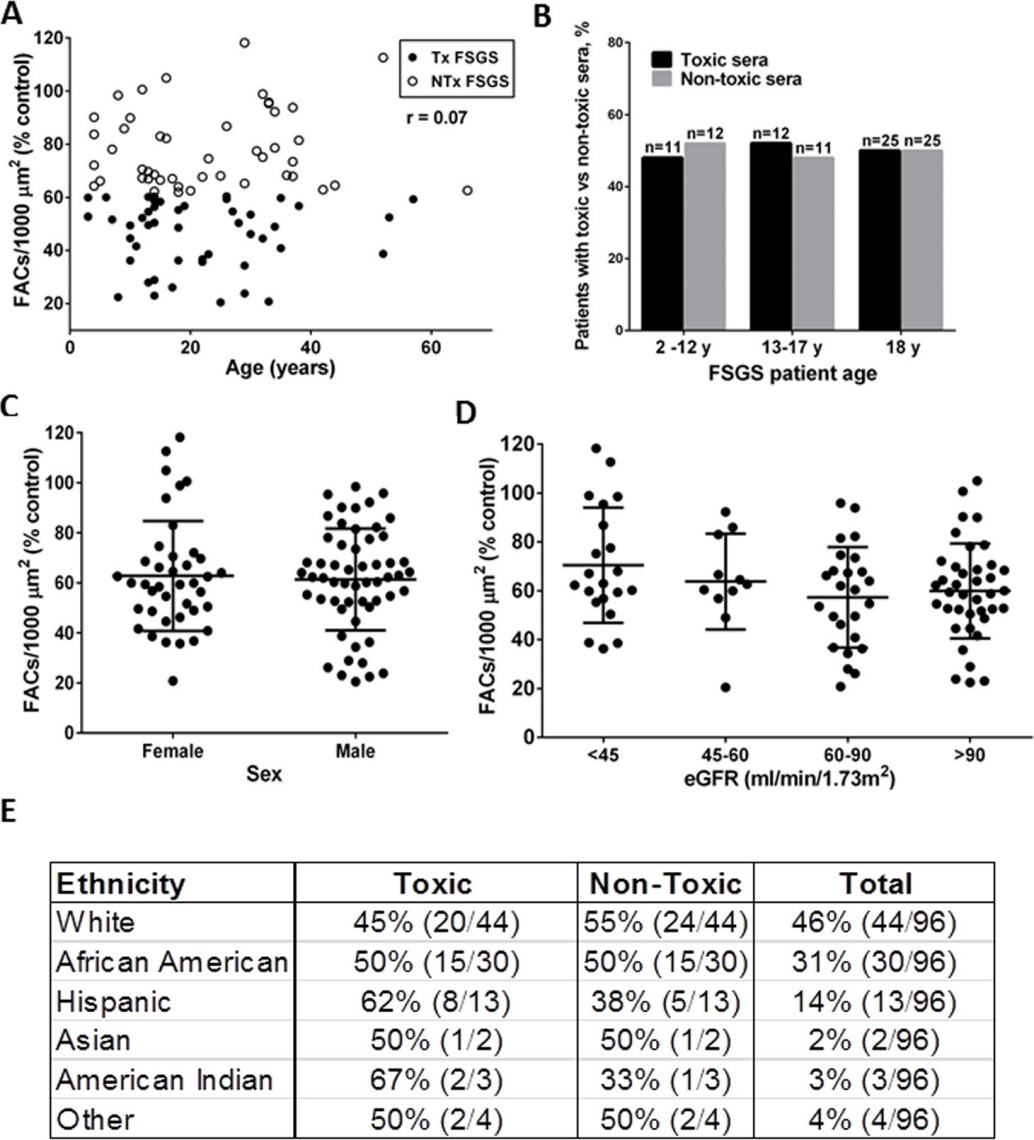
Correlation between in vitro toxicity and clinical parameters in the SRNS/FSGS cohort. (A) FAC numbers in cells treated with the SRNS/FSGS sera (N = 96) do not correlate with patient age. (B) Percentages of toxic vs non-toxic sera are stratified by age: 2–12 years, 13–17 years and 18 and older. Percentage of toxic vs non-toxic samples was calculated within each group. No statistically significant differences were detected. (C) FAC numbers in cells treated with sera of patients stratified by sex. No statistically significant differences were detected (means and standard deviations). (D) FAC numbers in the cells treated with sera of the patients stratified by eGFR. No statistically significant differences were detected (means and standard deviations). (E) Toxic podocyte serum activity in patients of various ethnicities.

The age of the entire cohort at the time of blood collection was 22±13 years. The age of the patients with serum podocyte-toxicity was similar to those without toxicity (21±13 years vs 22±14 years, respectively) (Fig 2A). Since it was suggested that idiopathic SRNS/FSGS may be more frequent among teenagers [31], we stratified patients into 3 categories: 2–12 years, 13–17 years and ≥18 years, based on the age at the time of blood collection. However, the percent of patients with toxic sera in the 13–17 age group (52%) was comparable to the other groups (Fig 2B). The presence of podocyte-toxic activity did not correlate with sex (Fig 2C), eGFR (Fig 2D) or proteinuria (S1 Fig).

We did not detect an increase in the frequency of serum podocyte toxicity in African Americans (50%) compared to Caucasians (55%) (Fig 2E). Interestingly, the percentage of samples showing podocyte toxicity was significantly elevated among Hispanics (62%) and American Indians (67%) (p<0.05 between toxic and non-toxic group for both ethnic groups), although the number of patients in these groups was low (Fig 2E).

Detailed kidney biopsy reports (light microscopy) were available in 11 patients in the MUHC cohort: segmental sclerosis ranged from 2–50% of glomeruli. Electron microscopy reports were available in nine patients: six biopsies showed extensive effacement of foot processes, one showed partial effacement, and there was minimal/no effacement in two. Seven of these nine patients received kidney transplants; FSGS recurred in three: all three showed extensive effacement of foot processes and the FAC assay ranged from 36–45% of control. Among four patients without recurrent FSGS, two had extensive effacement (FAC assay 87% (NPSH2 mutation) and 105%), one showed partial effacement (FAC assay 72%, PLCε1 mutation) and one showed no effacement (FAC assay 68%, INF2 mutation). 69 serum samples were from the FSGS-CT cohort; the FSGS pathology subtypes for the entire FSGS-CT cohort (N = 138) are described in [32].

### The TNFα signaling pathway mediates the toxicity of sera of certain FSGS patients

**Fig 3:**
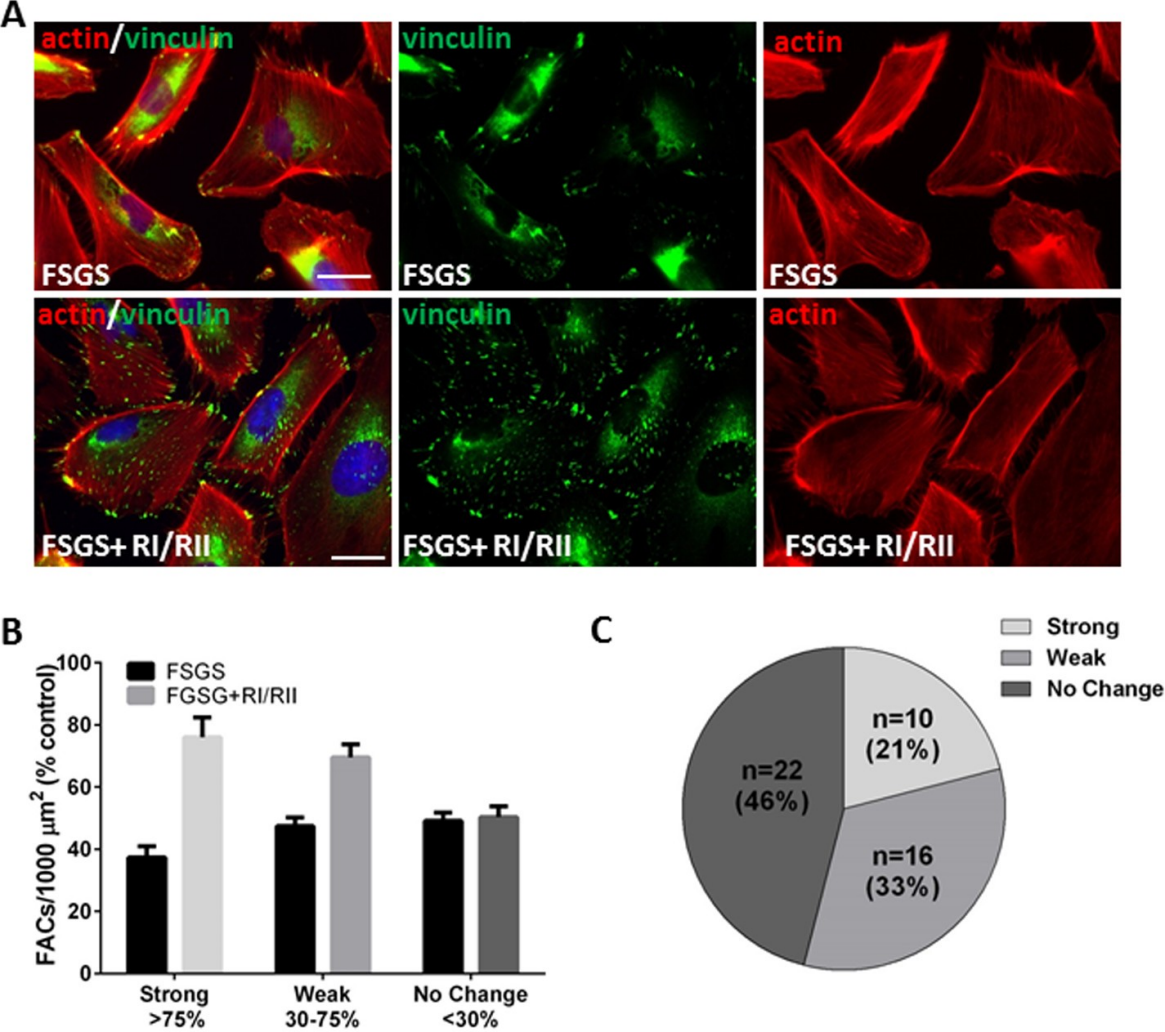
Inhibition of serum podocyte toxicity by blockade of TNFα signaling. (A) *Upper panels*—images of podocytes treated with 10% serum from a patient with podocyte toxic activity: FACs were immunostained with anti-vinculin antibody (green), actin was visualized with phalloidin (red) and nuclei were stained with DAPI. *Lower panels*: Images of podocytes pre-incubated with blocking antibodies against TNFα Receptor I and II (RI/II) prior to addition of the FSGS serum sample used in the images of the upper panel; scale bar 5 μm. (B) Effect of TNFα blockade on FAC numbers in podocytes exposed to toxic sera (N = 48). Means and standard errors are shown. (C) Percentages of patient serum samples with “strong” response (≥75% increase in the FAC number upon TNFα blockade vs no blockade for each sample tested), “weak” response (50% increase in the FAC numbers upon TNFα blockade vs no blockade for each sample tested) and “no response”.

We showed previously that blockade of TNFα signaling in podocytes *in vitro* significantly decreases podocyte toxic activity of some, but not all FSGS serum samples [22, 23]. The FONT I and II clinical trials using TNFα blockade (adalimumab) also showed heterogeneity in response among SRNS/FSGS patients [20, 21]. To evaluate if TNFα blockade attenuates podocyte toxicity of FSGS sera, we assayed sera that showed significant toxicity (N = 48, Fig 1C) in the presence or absence of anti-TNFα receptor I and II blocking antibodies ([23]; Fig 3). In 10/48 (21%) cases, serum toxicity was strongly suppressed by pretreatment of podocytes with these antibodies. The number of FACs increased 2-fold from mean 37.3% without TNFα blockade to 75.9% with blockade (Fig 3B and 3C, “strong” responders). In 16/48 (33%) cases, the response to TNFα blockade resulted in a ~50% increase in the number of FACs from mean 47.4% to 69.6% (“weak” responders). In 22/48 (46%) of samples, there was no change in the number of FACs: mean 49.1% without blockade vs 50.4% with blockade. Importantly, the serum toxicity of the patient with recurrent FSGS who favorably responded to TNFα blockade *in vivo* (previously described by our group in [22]) could be strongly suppressed by TNFα blockade in our FAC assay (S2A Fig).

### Podocyte toxic activity in serum is independent of serum TNFα level

**Fig 4:**
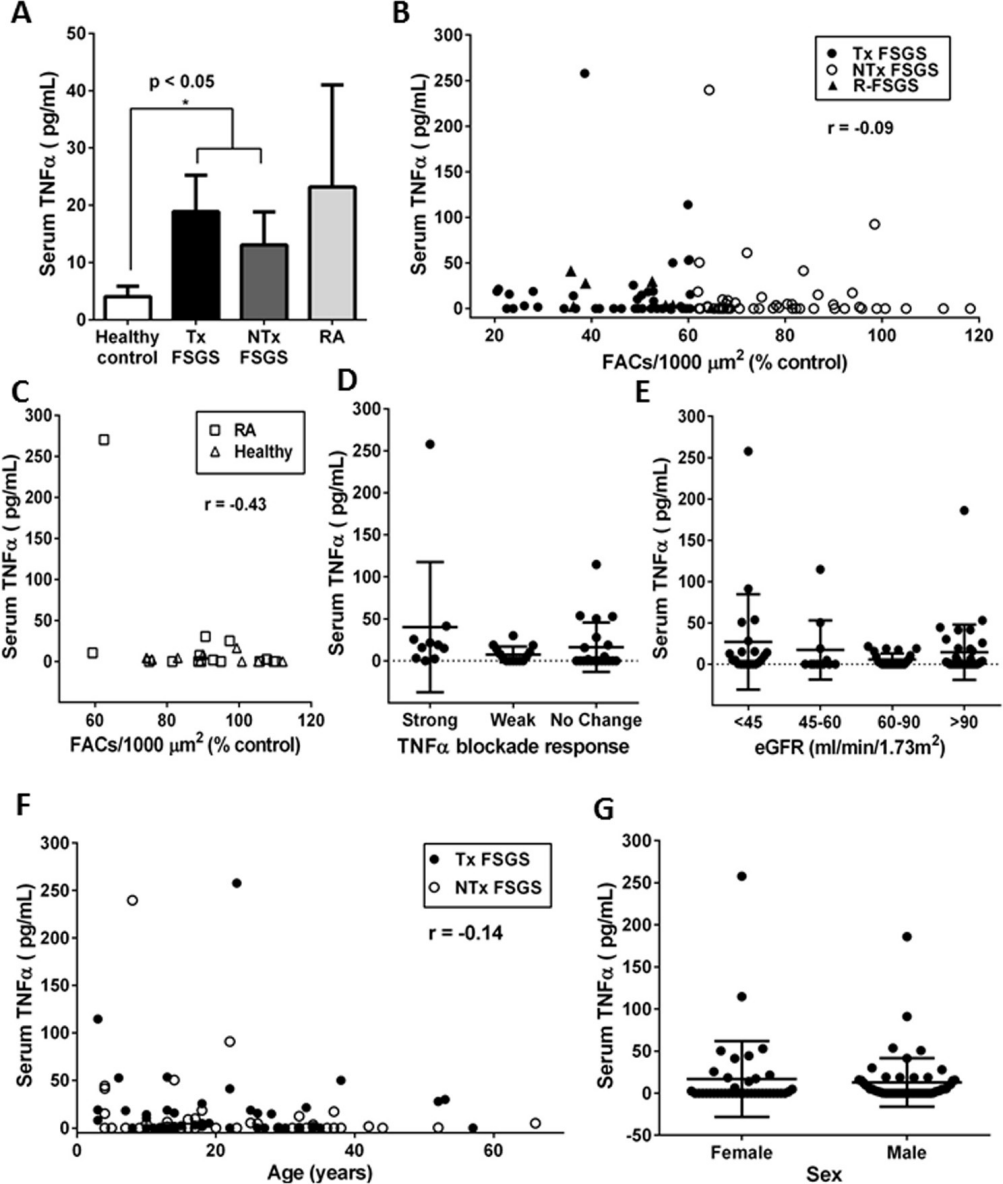
Correlation between serum TNFα levels, serum podocyte toxicity and clinical parameters. (A) Serum TNFα levels in healthy individuals (N = 9), SRNS/FSGS patients with toxic serum activity (FSGS-Tx, N = 45) and non-toxic sera (FSGS-NTx, N = 45), and patients with rheumatoid arthritis (N = 15); means and standard errors are shown. (B) FAC numbers and TNFα serum levels. (C) FAC numbers and TNFα serum levels in RA patients. (D) TNFα serum levels in the “strong” TNFα blockade responders, “weak” responders and “no response” samples (means and standard deviations). (E) TNFα serum levels in patients according to levels of eGFR (means and standard deviations). (F) TNFα serum levels and patient age. (G) Serum TNFα levels and sex (means and standard deviations).

A subset of patients with glomerular diseases, including FSGS, may have elevated serum levels of TNFα [15, 33]. We, therefore, measured TNFα in the FSGS serum samples. The mean serum TNFα in all SRNS/FSGS patients was 15.0 pg/ml, which was significantly higher than in the healthy controls (4.1 pg/ml), in keeping with several reports [14, 33]. However, the TNFα levels in sera with podocyte toxicity (18.5 pg/ml) were not statistically different from levels in non-toxic SRNS/FSGS sera (13.0 pg/ml) or RA sera (23.2 pg/ml) (Fig 4A). Furthermore, there was no correlation between FAC loss after exposure to toxic serum and the levels of serum TNFα (Fig 4B). Serum TNFα in recurrent FSGS patients (triangles) were not significantly different from non-toxic FSGS samples (open circles; Fig 4B). In some RA patients, we detected high levels of circulating TNFα; however, podocyte exposure to these sera did not cause FAC disassembly below 60% of untreated controls (Fig 4C).

Interestingly, the response to TNFα blockade did not correlate with serum TNFα levels (Fig 4D). Likewise, there was no association between eGFR and serum TNFα (Fig 4E), age (Fig 4F) or sex (Fig 4G). Taken together, we conclude that circulating TNFα does not contribute to podocyte toxicity of SRNS/FSGS serum and does not predict response to TNFα blockade.

### The TNFα pathway is activated in glomeruli of FSGS patients

**Fig 5:**
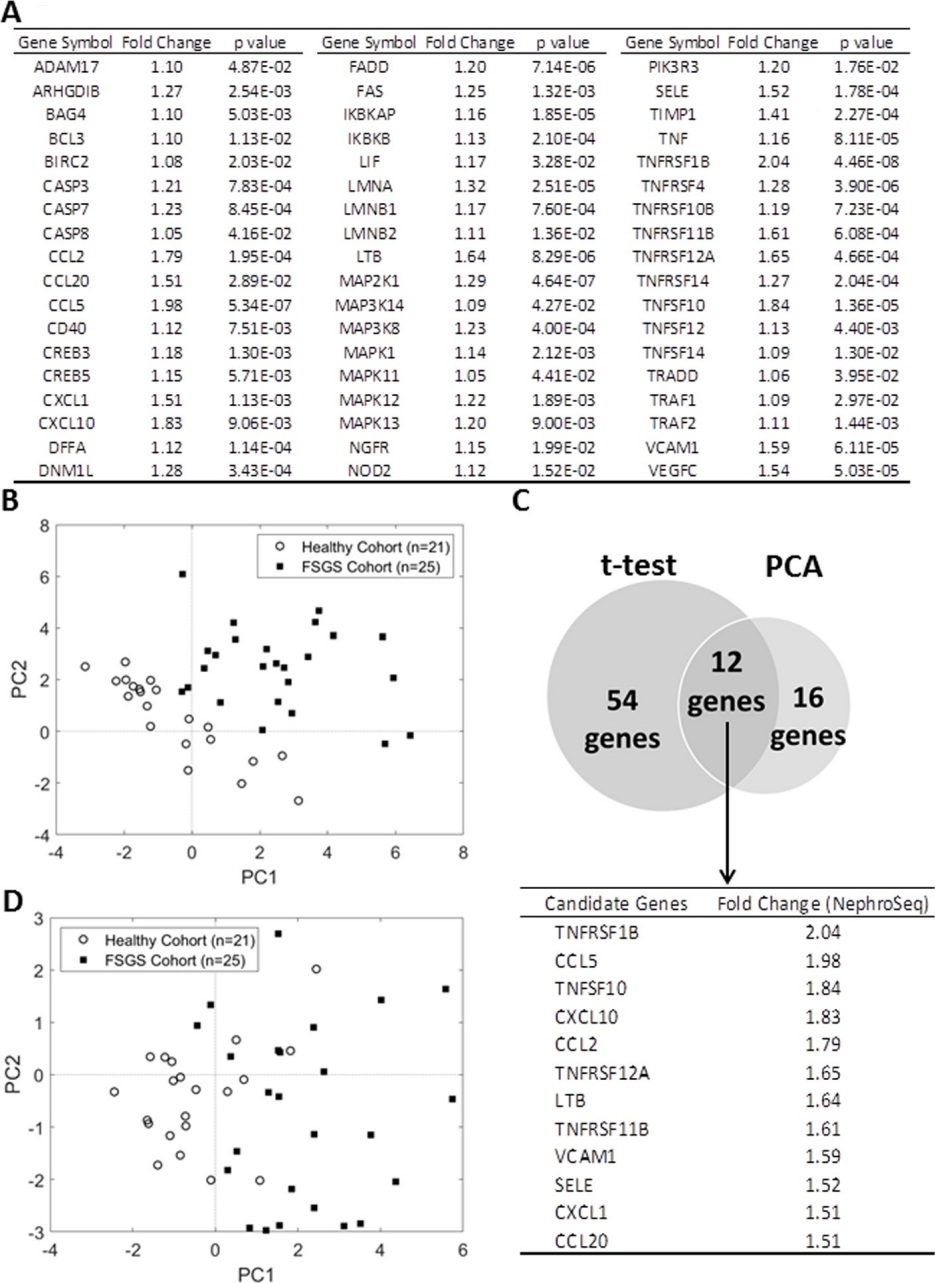
Analysis of TNFα gene expression in glomeruli of FSGS patients. (A) List of the TNFα pathway genes showing significantly greater expression in FSGS patients vs healthy controls in the Nephroseq dataset (t-test). (B) PCA of gene expression in the Nephroseq FSGSs cohort vs healthy controls. **C.** Venn diagram of the TNFα pathway genes showing both significantly greater expression (panel A; t-test) and contributing significantly to the PCA (panel B) in the FSGS patients vs healthy control subjects. (D) PCA of the 12 overlapping genes in panel C.

The results above suggest that expression of TNFα pathway genes in glomeruli may be altered in FSGS. Thus, we analyzed expression of TNFα pathway genes in a dataset from Nephroseq (S2 and S3 Tables). This dataset presents an analysis of mRNAs from laser-captured glomeruli of 25 patients with various stages of SRNS/FSGS vs 21 healthy individuals. Expression of 54/157 TNFα pathway genes was significantly upregulated in SRNS/FSGS patients compared to healthy controls (Fig 5A and S3 Fig) and these changes were detected in the FSGS samples across all levels of eGFR (S4 Fig), suggesting induction of an intrinsic TNFα pathway in the glomerulus that is independent of increased inflammation seen at late CKD stages [34].

We used principal component analysis (PCA) to compare the expression of the 157 TNFα pathway genes between individual FSGS patients and control individuals. PCA revealed that FSGS patients and controls could be separated into non-overlapping populations based on the TNFα pathway gene expression (Fig 5B). We then selected 16 genes that defined the greatest variance between the FSGS and control groups in the PCA, and combined the analysis of significant increases in individual genes (t-test; Fig 5A) with the genes that defined the greatest variance in the PCA (Fig 5B) to delineate a group of 12 overlapping candidate genes (Fig 5C). A separate PCA of only these 12 genes in each of the 25 patients vs 21 controls indicates that the differences in the expression of these 12 genes account for a nearly non-overlapping separation of the FSGS samples from the controls (Fig 5D).

### Sera from FSGS patients impact on TNFα gene expression in cultured podocytes

**Fig 6:**
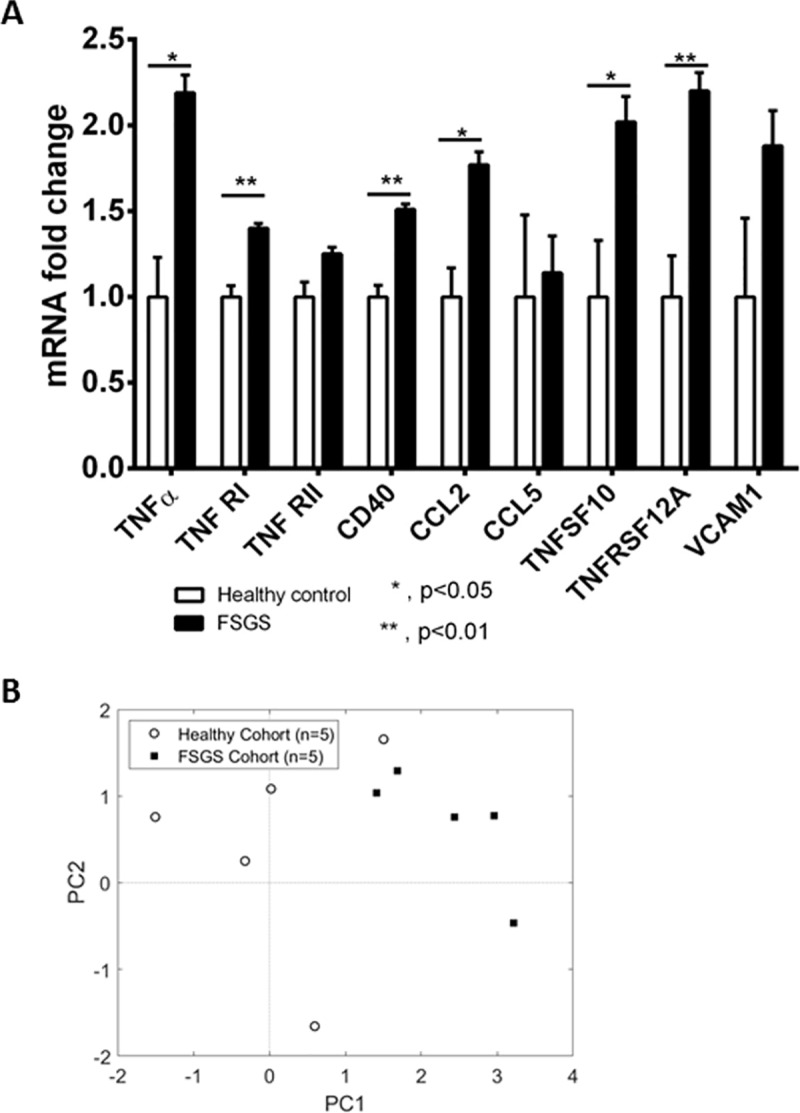
(A) RT-qPCR analysis of 9 TNFα pathway genes in cultured podocytes treated with five toxic SRNS/FSGS and five healthy control serum samples (means and standard errors). Experiments were repeated twice in duplicate for each serum sample (biological replicates), the amplification was carried out twice for each biological replicate in triplicate for each gene. (B) PCA of gene expression (9 genes) in cultured podocytes in response to FSGS sera vs healthy control sera.

Guided by the Nephroseq dataset analysis and previous studies [33, 35], we analyzed expression (RT-qPCR) of the genes that showed the greatest differences in the PCA analysis, as well as TNFα, TNFRI and CD40 in human podocytes incubated with five toxic SRNS/FSGS serum samples and five healthy control sera. Expression of 6/9 tested genes was elevated significantly by SRNS/FSGS sera, compared to healthy sera (Fig 6A). The remaining 3 transcripts tended to be elevated, but changes did not reach statistical significance due to high variability among serum-treated cells, and low basal levels of expression in podocytes. The PCA for the five patient and five control samples using expression data for 9 tested by qPCR genes revealed a clear non-overlapping separation between the FSGS group and healthy controls (Fig 6B). Overall, our data are in support of a substantial elevation in TNFα pathway gene expression in podocytes in response to FSGS sera *in vitro* and in the context of disease *in vivo*. Importantly, the serum of the index patient treated with infliximab/etanercept *in vivo* [22] triggered similar upregulation of the TNFα pathway genes in cultured podocytes (S2B Fig).

## Discussion

In the present study, we have shown that an image-based *in vitro* assay can identify patients whose sera contain a podocyte-toxic activity, and who may be at risk for developing recurrent FSGS post-transplant. The assay also identifies those patients in whom serum podocyte toxicity may be reversed by TNFα blockade. Moreover, we have shown that activation of TNFα pathway genes occurs in glomeruli of patients with FSGS.

First, the screening assay identified those FSGS patients who exhibit circulating podocyte-toxic activity (disassembly of FACs) and distinguished between the recurrent/idiopathic FSGS patients (this manuscript and [23]) and non-recurrent post-transplant patients or patients with genetic causes of SRNS/FSGS [24]. The present study also established the prevalence of a circulating podocyte toxic factor in SRNS/FSGS patients, i.e. in 48/96 (50%) of SRNS/FSGS samples analyzed. Depending on the cohort, post-transplant recurrence has been reported in 20–60% of idiopathic SRNS/FSGS patients [1, 31, 36], and all 7 patients in our cohort with proven recurrence showed serum podocyte toxicity. Considering the large proportion of younger patients in our tested cohort (younger than age 18, N = 46), and young age being a known risk factor for recurrence [31], the percentage of samples with detected toxic activity (50%) is in keeping with published reports. About 20–40% of pediatric SRNS/FSGS patients harbor pathogenic mutations; the prevalence of mutations in very young children is high (~ 50–70% in SRNS children under 1 year of age) [6], while in children older than age 1 and in adult patients is much lower [4, 5]. The majority of our patients were not tested for podocyte gene mutations (a limitation of our study); however, given the age of our cohort (2–62 years at the time of onset, except for 6 younger patients in the MUHC cohort), it is unlikely that gene mutations account for all patients lacking serum podocyte toxicity. Hence, it is conceivable that our assay identifies most patients at risk for recurrent disease, yet there is likely a third subset of SRNS/FSGS patients who lack known genetic mutations, but are not at risk for recurrence in the renal allograft. Although reports of glomerular ultrastructure were available in only a small number of patients, we could conclude that all patients with recurrent FSGS showed both loss of FACs below 60% and extensive foot process effacement, signifying a correlation between diffuse foot process effacement and presence of a circulating toxic factor.

Elevated levels of pro-inflammatory cytokines including TNFα have been reported in the general CKD population and in end stage renal disease [34, 37]. However, the relationship between serum TNFα and SRNS/FSGS is controversial. Kacprzyk reported elevated serum TNFα in various glomerulopathies including FSGS, but the number of tested patients was low [15]. Syranyi *et al* detected elevated TNFα plasma levels in 11 of 17 FSGS patients; the TNFα levels did not correlate with age, sex, severity of proteinuria or level of renal impairment [14]. Interestingly, Pedigo *et al* recently reported no correlation between TNFα serum levels and adverse effects of SRNS/FSGS sera on podocyte viability [17]. The present study shows that serum TNFα is significantly elevated in 96 FSGS patients compared to healthy controls (Fig 3); however, the levels of TNFα do not correlate with serum podocyte toxicity in the FAC assay or renal impairment (eGFR). In Pedigo’s report, 6 patients with FSGS and 14 patients with steroid-dependent nephrotic syndrome were found to have levels of serum TNFα comparable to healthy controls. Collectively, these observations argue against TNFα as being the podocyte toxic factor in SRNS/FSGS serum. Consistent with this conclusion, we did not detect increased podocyte-toxic activity in RA sera (mean serum TNFα was even higher than in the toxic FSGS group).

Our observations implicate an *intrinsic* podocyte TNFα signaling pathway in the pathogenesis of SRNS/FSGS. Incubation of rat glomerular isolate with TNFα increases glomerular albumin permeability due to generation of reactive oxidative species [38], and infusion of TNFα in mice causes foot process effacement and proteinuria [17]. We previously showed that exposure of cultured podocytes to TNFα leads to dramatic changes in actin arrangement and a loss of FACs similar to the effects of toxic sera from FSGS patients [23], suggesting that activation of TNFα signaling in podocytes affects the cytoskeleton and adhesion. However, in most experimental conditions, the concentration of recombinant TNFα used (0.4–100 ng/ml) was significantly higher than found in the serum of patients in the present study. Binding of TNFα to its two receptors, TNFRI and TNFRII, de-represses the critical TNFα effector, nuclear factor kappa-light-chain-enhancer of activated B cells (NF-κB), which translocates to the nucleus and activates a variety of downstream targets. Importantly, Hussain *et al* showed that constitutive activation of NF-κB in mouse podocytes *in vivo* leads to proteinuria accompanied by foot process effacement and podocyte loss [39]. Moreover, the authors found that in nephrotic syndrome due to NPHS1 mutations, nephrin deficiency activates the intrinsic NF-κB pathway in podocytes promoting podocyte injury [39]. Taken together, activation of TNFα signaling appears to cause and/or increase podocyte injury in various contexts.

TNFα blockade reversed SRNS/FSGS serum podocyte toxicity in ~21% of the toxic samples. The FONT I/II study found that 4/17 (23.5%) of patients responded to TNFα blockade (adalimumab) *in vivo*. Thus, our observations parallel the FONT I/II study and indicate heterogeneity among SRNS/FSGS patients in regard to TNFα blockade responsiveness. A plausible explanation is that FSGS is due to activation of the intrinsic TNFα podocyte pathway in some patients, while alternative pathways may drive podocyte injury in others. We propose that among patients with serum podocyte toxic activity, response to TNFα blockade *in vitro* may predict patient responsiveness to anti-TNFα drugs *in vivo*. Interestingly, the effect of SRNS/FSGS serum on TNFα pathway-induced podocyte FAC loss was independent of serum TNFα level, as noted above. Moreover, serum TNFα levels were not associated with responsiveness to TNFα blockade *in vitro*. These results imply that podocyte injury is due to the activation of an intrinsic podocyte TNFα signaling pathway. Using Nephroseq dataset, we evaluated expression of the 157 TNFα pathway genes in the glomerular mRNA from biopsies of 25 FSGS patients vs 21 controls. Our analysis revealed a robust upregulation of numerous TNFα pathway genes in the biopsies of FSGS patients. Remarkably, the PCA analysis demonstrated in an unbiased fashion that the FSGS patients can be separated from controls solely on the basis of upregulated expression of the TNFα genes. These results were validated experimentally in podocytes treated with SRNS/FSGS sera. Our observations expand on an earlier study that showed upregulation of a more limited set of TNFα pathway genes with FSGS sera [17] and substantiate the view that the TNFα signaling pathway is an important downstream effector of the circulating toxic factor in podocytes *in vivo* and *in vitro*.

In summary, we have confirmed that cases of idiopathic SRNS/FSGS can be identified by conducting a functional assay in cultured human podocytes that assesses the extent of FAC disassembly. We also found that in approximately a quarter of the patients with confirmed serum podocyte toxicity *in vitro*, the toxic serum effects can be reversed by TNFα blockade and that these sera increase expression of TNFα pathway mRNAs in cultured podocytes. We propose that *in vitro* analyses of patient sera can help establish the mechanistic pathways of podocyte injury in FSGS, and stratify patients with regards to their potential responses to specific clinical interventions.

## Supporting information

S1 Table PCR primers. (PDF)

S2 Table TNFα pathway gene query. (PDF)

S3 Table Clinical data of 25 FSGS patients in the “Ju CKD Glom” dataset. (PDF)

S1 Fig Relationship between FACs and proteinuria. (TIF)

S2 Fig Effect of serum from a recurrent FSGS patient successfully treated with etanercept/infliximab on FACs and TNFα pathway gene expression in cultured podocytes. (TIF)

S3 Fig Heat Map of the TNFα pathway genes in FSGS patients vs controls. (TIF)

S4 Fig TNFα pathway gene expression does not depend on stage of chronic kidney disease. (TIF)
