# Supplementary material for: Intrinsic tumor necrosis factor-α pathway is activated in a subset of patients with focal segmental glomerulosclerosis
Source: PLoS One. 2019 May 16;14(5):e0216426. doi: 10.1371/journal.pone.0216426 (PMC6522053; doi:10.1371/journal.pone.0216426)
Supplement: S1 Table — (PDF) [file pone.0216426.s001.pdf]

S1 Table

Amplification parameters were: 95°C for 5 min, followed by 40 cycles of 5 s at 95°C, 10 s at 65°C and 10 s at 72°C, followed by 5 s at 95°C.

huTNF-F: CAACCTCCTCTCTGCCATCAAGA
huTNF-R: CTGGAAGACCCCTCCCAGATAGA
huTNFRSF1A-F (TNFRI): GTGCCTACCCCAGATTGAGA
huTNFRSF1A-R (TNFRI): TGTCGATTTCCCACAAACAA
huTNFRSF1B-F (TNFRII): GGAAACTCAAGCCTGCACTC
huTNFRSF1B-R (TNFRII): TGCAAATATCCGTGGATGAA
huCCL2-F: CCCCAGTCACCTGCTGTTAT
huCCL2-R: AGATCTCCTTGGCCACAATG
huCCL5-F: GAGGCTTCCCCTCACTATCC
huCCL5-R: CTCAAGTGATCCACCCACCT
huCD40-F: GCAGGCACAAACAAGACTGA
huCD40-R: TCGGGAAAATTGATCTCCTG
huVCAM-F: CTACGCTGACAATGAATCCTGTT
huVCAM-R: CCAGAGGGCCACTCAAATGAA
huTNFSF10-F: TCAAGTGGCAACTCCGTCAG
huTNFSF10-R: TGGTCCCAGTTATGTGAGCTG
huTNFRSF12A-F: CTCTGAGCCTGACCTTCGTG
huTNFRSF12A-R: GGGGCACATTGTCACTGGAT
