# Supplementary material for: Intrinsic tumor necrosis factor-α pathway is activated in a subset of patients with focal segmental glomerulosclerosis
Source: PLoS One. 2019 May 16;14(5):e0216426. doi: 10.1371/journal.pone.0216426 (PMC6522053; doi:10.1371/journal.pone.0216426)
Supplement: S2 Table — (PDF) [file pone.0216426.s002.pdf]

S2 Table 2

	TNFa gene pathway query - list	
#	**Gene Name**	**Gene Symbol**
1	ArfGAP with FG repeats 1	AGFG1
2	ArfGAP with FG repeats 2	AGFG2
3	v-akt murine thymoma viral oncogene homolog 1	AKT1
4	v-akt murine thymoma viral oncogene homolog 2	AKT2
5	v-akt murine thymoma viral oncogene homolog 3 (protein kinase B, gamma)	AKT3
6	Rho GDP dissociation inhibitor (GDI) beta	ARHGDIB
7	activating transcription factor 2	ATF2
8	activating transcription factor 6 beta	ATF6B
9	BCL2-associated athanogene 4	BAG4
10	B-cell CLL/lymphoma 3	BCL3
11	baculoviral IAP repeat-containing 2	BIRC2
12	baculoviral IAP repeat-containing 3	BIRC3
13	carbamoyl-phosphate synthetase 2, aspartate transcarbamylase, and dihydroorotase	CAD
14	caspase 10, apoptosis-related cysteine peptidase	CASP10
15	caspase 2, apoptosis-related cysteine peptidase	CASP2
16	caspase 3, apoptosis-related cysteine peptidase	CASP3
17	caspase 7, apoptosis-related cysteine peptidase	CASP7
18	caspase 8, apoptosis-related cysteine peptidase	CASP8
19	chemokine (C-C motif) ligand 2	CCL2
20	chemokine (C-C motif) ligand 20	CCL20
21	chemokine (C-C motif) ligand 5	CCL5
22	CD27 molecule	CD27
23	CD40 molecule, TNF receptor superfamily member 5	CD40
24	CD40 ligand	CD40LG
25	CD70 molecule	CD70
26	CCAAT/enhancer binding protein (C/EBP), beta	CEBPB
27	CASP8 and FADD-like apoptosis regulator	CFLAR
28	conserved helix-loop-helix ubiquitous kinase	CHUK
29	CASP2 and RIPK1 domain containing adaptor with death domain	CRADD
30	cAMP responsive element binding protein 1	CREB1
31	cAMP responsive element binding protein 3	CREB3
32	cAMP responsive element binding protein 3-like 1	CREB3L1
33	cAMP responsive element binding protein 3-like 2	CREB3L2
34	cAMP responsive element binding protein 5	CREB5
35	colony stimulating factor 1 (macrophage)	CSF1
36	colony stimulating factor 2 (granulocyte-macrophage)	CSF2
37	chemokine (C-X3-C motif) ligand 1	CX3CL1
38	chemokine (C-X-C motif) ligand 1 (melanoma growth stimulating activity, alpha)	CXCL1
39	chemokine (C-X-C motif) ligand 10	CXCL10
40	chemokine (C-X-C motif) ligand 2	CXCL2
41	chemokine (C-X-C motif) ligand 3	CXCL3
42	chemokine (C-X-C motif) ligand 5	CXCL5
43	DNA fragmentation factor, 45kDa, alpha polypeptide	DFFA
44	dynamin 1-like	DNM1L
45	dual specificity phosphatase 1	DUSP1

46	ectodysplasin A2 receptor	EDA2R
47	endothelin 1	EDN1
48	Fas (TNFRSF6)-associated via death domain	FADD
49	Fas (TNF receptor superfamily, member 6)	FAS
50	Fas ligand (TNF superfamily, member 6)	FASLG
51	FBJ murine osteosarcoma viral oncogene homolog	FOS
52	intercellular adhesion molecule 1	ICAM1
53	inhibitor of kappa light polypeptide gene enhancer in B-cells, kinase complex-associated protei	IKBKAP
54	inhibitor of kappa light polypeptide gene enhancer in B-cells, kinase beta	IKBKB
55	interleukin 15	IL15
56	interleukin 18 receptor 1	IL18R1
57	interleukin 1, beta	IL1B
58	interleukin 6 (interferon, beta 2)	IL6
59	itchy E3 ubiquitin protein ligase homolog (mouse)	ITCH
60	jagged 1 (Alagille syndrome)	JAG1
61	jun oncogene	JUN
62	jun B proto-oncogene	JUNB
63	leukemia inhibitory factor (cholinergic differentiation factor)	LIF
64	lamin A/C	LMNA
65	lamin B1	LMNB1
66	lamin B2	LMNB2
67	lymphotoxin alpha (TNF superfamily, member 1)	LTA
68	lymphotoxin beta (TNF superfamily, member 3)	LTB
69	lymphotoxin beta receptor (TNFR superfamily, member 3)	LTBR
70	MAP-kinase activating death domain	MADD
71	membrane associated guanylate kinase, WW and PDZ domain containing 2	MAGI2
72	mitogen-activated protein kinase kinase 1	MAP2K1
73	mitogen-activated protein kinase kinase 3	MAP2K3
74	mitogen-activated protein kinase kinase 4	MAP2K4
75	mitogen-activated protein kinase kinase 6	MAP2K6
76	mitogen-activated protein kinase kinase 7	MAP2K7
77	mitogen-activated protein kinase kinase kinase 1	MAP3K1
78	mitogen-activated protein kinase kinase kinase 14	MAP3K14
79	mitogen-activated protein kinase kinase kinase 5	MAP3K5
80	mitogen-activated protein kinase kinase kinase 7	MAP3K7
81	mitogen-activated protein kinase kinase kinase 8	MAP3K8
82	mitogen-activated protein kinase 1	MAPK1
83	mitogen-activated protein kinase 10	MAPK10
84	mitogen-activated protein kinase 11	MAPK11
85	mitogen-activated protein kinase 12	MAPK12
86	mitogen-activated protein kinase 13	MAPK13
87	mitogen-activated protein kinase 14	MAPK14
88	mitogen-activated protein kinase 3	MAPK3
89	mitogen-activated protein kinase 8	MAPK8
90	mitogen-activated protein kinase 9	MAPK9
91	matrix metallopeptidase 14 (membrane-inserted)	MMP14
92	matrix metallopeptidase 3 (stromelysin 1, progelatinase)	MMP3
93	matrix metallopeptidase 9 (gelatinase B, 92kDa gelatinase, 92kDa type IV collagenase)	MMP9

94	nuclear factor of kappa light polypeptide gene enhancer in B-cells 1	NFKB1
95	nuclear factor of kappa light polypeptide gene enhancer in B-cells inhibitor, alpha	NFKBIA
96	nerve growth factor receptor	NGFR
97	nucleotide-binding oligomerization domain containing 2	NOD2
98	p21 protein (Cdc42/Rac)-activated kinase 1	PAK1
99	p21 protein (Cdc42/Rac)-activated kinase 2	PAK2
100	poly (ADP-ribose) polymerase 1	PARP1
101	peptidoglycan recognition protein 1	PGLYRP1
102	phosphoinositide-3-kinase, catalytic, alpha polypeptide	PIK3CA
103	phosphoinositide-3-kinase, catalytic, beta polypeptide	PIK3CB
104	phosphoinositide-3-kinase, catalytic, delta polypeptide	PIK3CD
105	phosphoinositide-3-kinase, catalytic, gamma polypeptide	PIK3CG
106	phosphoinositide-3-kinase, regulatory subunit 1 (alpha)	PIK3R1
107	phosphoinositide-3-kinase, regulatory subunit 2 (beta)	PIK3R2
108	phosphoinositide-3-kinase, regulatory subunit 3 (gamma)	PIK3R3
109	phosphoinositide-3-kinase, regulatory subunit 5	PIK3R5
110	protein kinase, DNA-activated, catalytic polypeptide	PRKDC
111	proteasome (prosome, macropain) assembly chaperone 2	PSMG2
112	prostaglandin-endoperoxide synthase 2 (prostaglandin G/H synthase and cyclooxygenase)	PTGS2
113	retinoblastoma 1	RB1
114	v-rel reticuloendotheliosis viral oncogene homolog A (avian)	RELA
115	receptor (TNFRSF)-interacting serine-threonine kinase 1	RIPK1
116	ribosomal protein S6 kinase, 90kDa, polypeptide 4	RPS6KA4
117	ribosomal protein S6 kinase, 90kDa, polypeptide 5	RPS6KA5
118	selectin E	SELE
119	suppressor of cytokine signaling 3	SOCS3
120	spectrin, alpha, non-erythrocytic 1 (alpha-fodrin)	SPTAN1
121	TGF-beta activated kinase 1/MAP3K7 binding protein 1	TAB1
122	TGF-beta activated kinase 1/MAP3K7 binding protein 2	TAB2
123	tumor necrosis factor	TNF
124	tumor necrosis factor, alpha-induced protein 3	TNFAIP3
125	tumor necrosis factor receptor superfamily, member 10b	TNFRSF10B
126	tumor necrosis factor receptor superfamily, member 10c, decoy without an intracellular domai	TNFRSF10C
127	tumor necrosis factor receptor superfamily, member 10d, decoy with truncated death domain	TNFRSF10D
128	tumor necrosis factor receptor superfamily, member 11a, NFKB activator	TNFRSF11A
129	tumor necrosis factor receptor superfamily, member 11b	TNFRSF11B
130	tumor necrosis factor receptor superfamily, member 12A	TNFRSF12A
131	tumor necrosis factor receptor superfamily, member 13B	TNFRSF13B
132	tumor necrosis factor receptor superfamily, member 14 (herpesvirus entry mediator)	TNFRSF14
133	tumor necrosis factor receptor superfamily, member 17	TNFRSF17
134	tumor necrosis factor receptor superfamily, member 1A	TNFRSF1A
135	tumor necrosis factor receptor superfamily, member 1B	TNFRSF1B
136	tumor necrosis factor receptor superfamily, member 21	TNFRSF21
137	tumor necrosis factor receptor superfamily, member 25	TNFRSF25
138	tumor necrosis factor receptor superfamily, member 4	TNFRSF4
139	tumor necrosis factor receptor superfamily, member 6b, decoy	TNFRSF6B
140	tumor necrosis factor receptor superfamily, member 8	TNFRSF8
141	tumor necrosis factor receptor superfamily, member 9	TNFRSF9

142	tumor necrosis factor (ligand) superfamily, member 10	TNFSF10
143	tumor necrosis factor (ligand) superfamily, member 11	TNFSF11
144	tumor necrosis factor (ligand) superfamily, member 12	TNFSF12
145	tumor necrosis factor (ligand) superfamily, member 14	TNFSF14
146	tumor necrosis factor (ligand) superfamily, member 15	TNFSF15
147	tumor necrosis factor (ligand) superfamily, member 18	TNFSF18
148	tumor necrosis factor (ligand) superfamily, member 4	TNFSF4
149	tumor necrosis factor (ligand) superfamily, member 8	TNFSF8
150	tumor necrosis factor (ligand) superfamily, member 9	TNFSF9
151	TNFRSF1A-associated via death domain	TRADD
152	TNF receptor-associated factor 1	TRAF1
153	TNF receptor-associated factor 2	TRAF2
154	TNF receptor-associated factor 3	TRAF3
155	TNF receptor-associated factor 5	TRAF5
156	vascular cell adhesion molecule 1	VCAM1
157	vascular endothelial growth factor C	VEGFC
