# Supplementary material for: Intrinsic tumor necrosis factor-α pathway is activated in a subset of patients with focal segmental glomerulosclerosis
Source: PLoS One. 2019 May 16;14(5):e0216426. doi: 10.1371/journal.pone.0216426 (PMC6522053; doi:10.1371/journal.pone.0216426)
Supplement: S3 Table — (PDF) [file pone.0216426.s003.pdf]

S3 Table

FSGS group	Cohort Number	Sex	Age (Years)	Creatinine, mg/dL	BUN (mg/dL)	eGFR (MDRD), mg/min/1.73m	CKD stage
Ju CKD dataset	1	M	53	1.2	23	67	2
	2	M	67	1.1	33	73	2
	3	M	N.A.	0.8	N.A.	N.A.	N.A.
	4	M	22	4.0	44	20	4
	5	F	62	3.2	86	21	4
	6	M	66	1.4	14	54	3A
	7	M	49	1.4	51	57	3A
	8	M	22	0.8	30	128	1
	9	M	37	2.0	65	40	3B
	10	F	66	1.3	45	44	3B
	11	M	40	0.9	N.A.	99	1
	12	M	32	1.0	20	92	1
	13	F	51	0.7	21	94	1
	14	F	67	0.8	13	80	2
	15	F	33	0.7	46	105	1
	16	F	47	1.1	17	60	2
	17	F	39	2.7	194	21	4
	18	M	32	0.8	36	119	1
	19	F	55	0.6	N.A.	111	1
	20	M	28	0.8	N.A.	125	1
	21	F	54	0.8	23	79	2
	22	F	21	0.7	7	112	1
	23	F	63	1.3	13	44	3B
	24	F	51	0.8	14	82	2
	25	M	43	1.4	17	60	2

List of abbreviations: BUN – blood urea nitrogen, eGFR – estimated glomerular filtration rate, MDRD -- Modification of Diet in Renal Disease Study formula to calculate eGFR, CKD – chronic kidney disease, M-male, F – female, N.A. – not available
