# Supplementary figures and images for: Intrinsic tumor necrosis factor-α pathway is activated in a subset of patients with focal segmental glomerulosclerosis
Source: PLoS One. 2019 May 16;14(5):e0216426. doi: 10.1371/journal.pone.0216426 (PMC6522053; doi:10.1371/journal.pone.0216426)

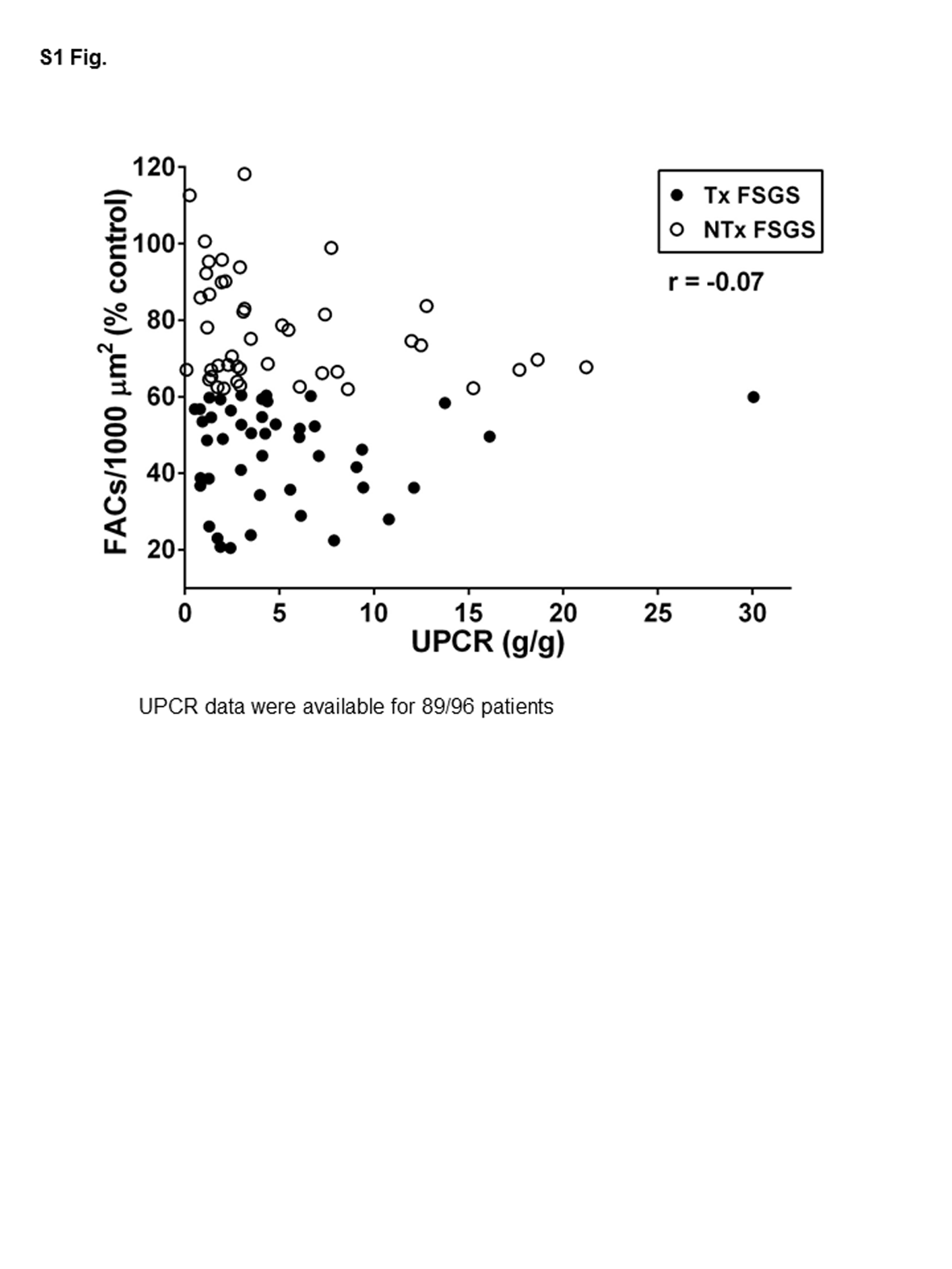

Supplement: S1 Fig — (TIF) [file pone.0216426.s004.tif]

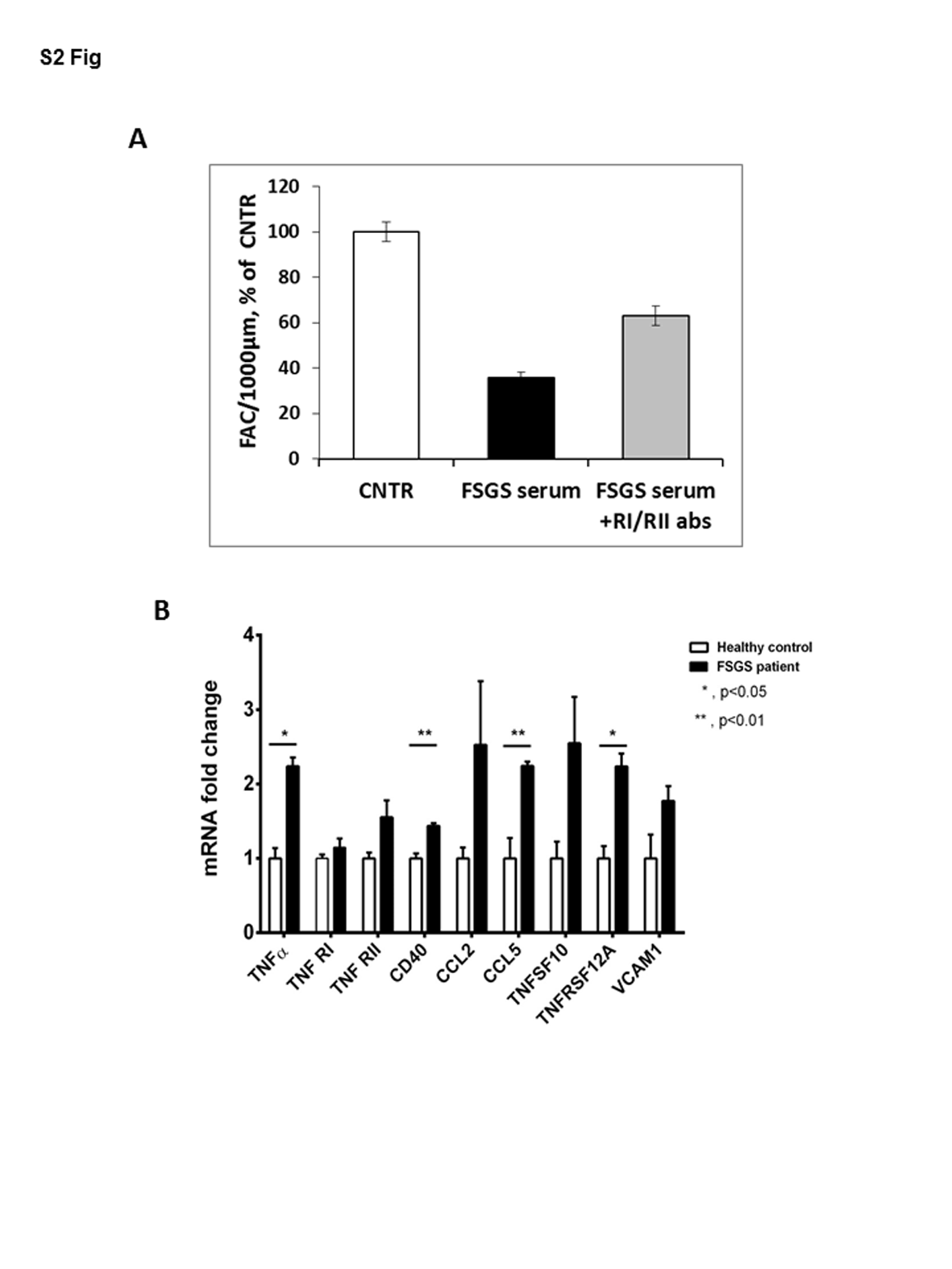

Supplement: S2 Fig — (TIF) [file pone.0216426.s005.tif]

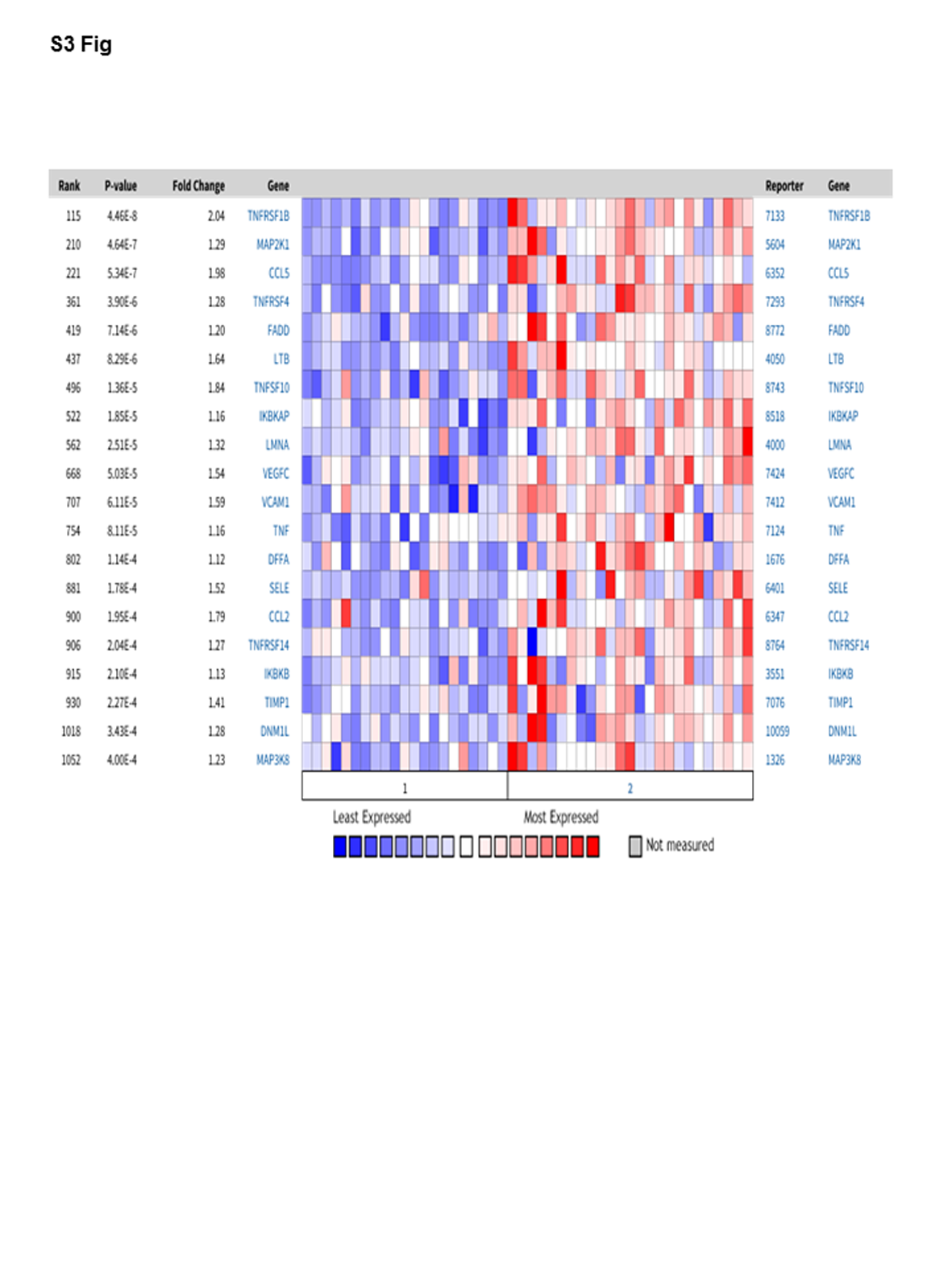

Supplement: S3 Fig — (TIF) [file pone.0216426.s006.tif]

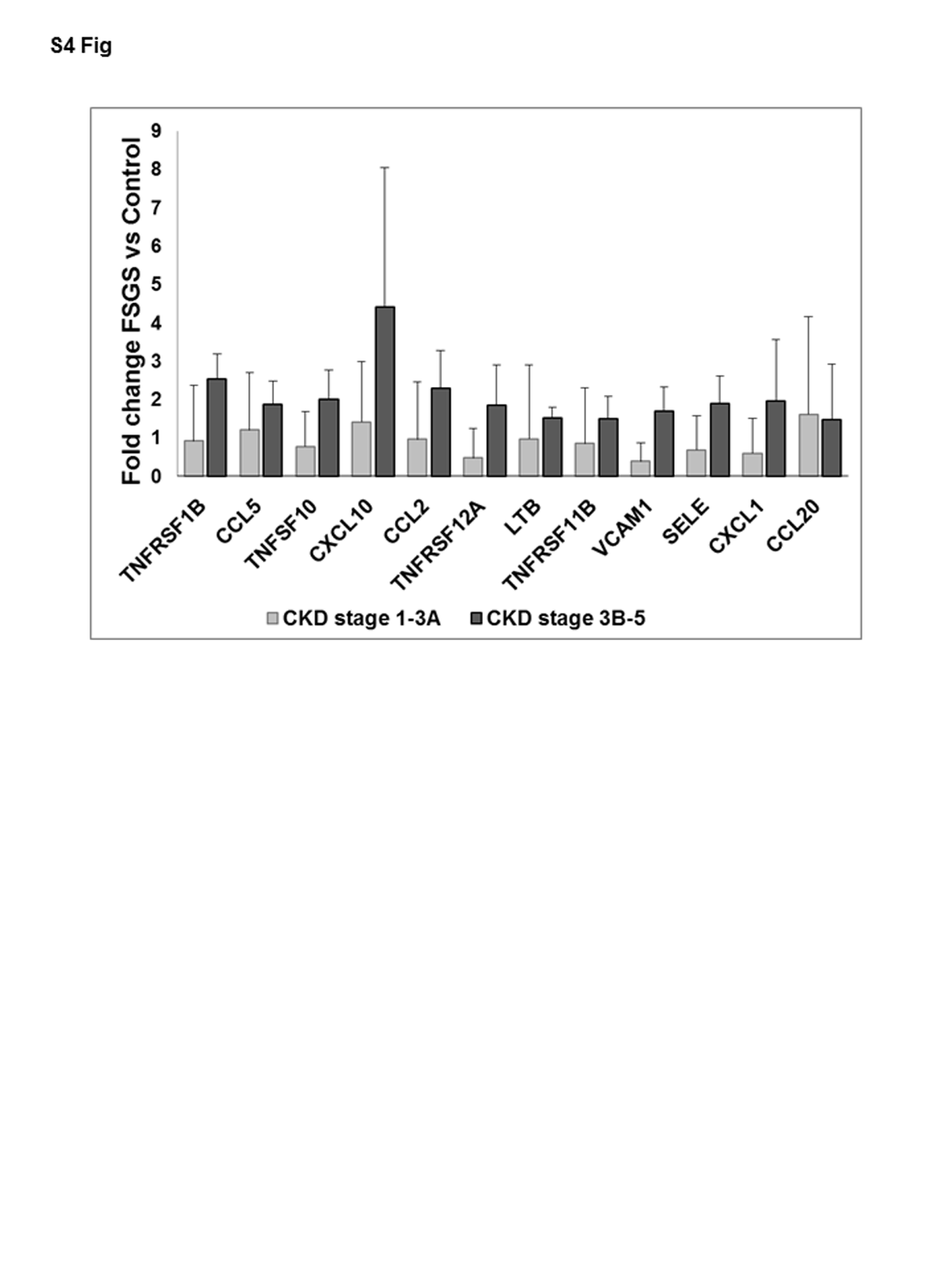

Supplement: S4 Fig — (TIF) [file pone.0216426.s007.tif]
